# Sero- and Feco-Prevalence of *Helicobacter pylori* Infection and Its Associated Risk Factors among Adult Dyspeptic Patients Visiting the Outpatient Department of Adet Primary Hospital, Yilmana Densa District, Northwest Ethiopia

**DOI:** 10.1155/2023/2305681

**Published:** 2023-07-17

**Authors:** Zebasil Mnichil, Endalkachew Nibret, Daniel Mekonnen, Maritu Demelash

**Affiliations:** ^1^Yilmana Densa Woreda Education Office, West Gojjam Zone, Bahir Dar, Ethiopia; ^2^Department of Biology, College of Science, Bahir Dar University, Bahir Dar, Ethiopia; ^3^Institute of Biotechnology (IOB), Bahir Dar University, Bahir Dar, Ethiopia; ^4^Department of Medical Laboratory Sciences, College of Medicine and Health Sciences, Bahir Dar University, Bahir Dar, Ethiopia; ^5^North Mecha Woreda Education Office, West Gojjam Zone, Bahir Dar, Ethiopia

## Abstract

**Background:**

Most gastric cancers in the world are attributed to *Helicobacter pylori* (*H. pylori*) infections. The prevalence of *H. pylori* infection is influenced by socioeconomic status, hygiene, and lifestyle of the population. This study aimed to assess sero- and feco-prevalence of *H. pylori* infections and its associated risk factors among adult dyspeptic patients visiting the outpatient department of Adet Primary Hospital, Yilmana Densa District, northwest Ethiopia.

**Methods:**

A hospital-based cross-sectional study was conducted from February 10, 2022 to April 10, 2022. The study participants were selected using a systematic random sampling technique. The data were collected by pretested questionnaires. Blood and stool specimens were collected from each patient for antibody and *H. pylori* antigen tests, respectively. The data were analyzed using Statistical Package for Social Science (SPSS) version 26.0. A logistic regression model was used to assess the strength of association between *H. pylori* positivity and risk factors.

**Results:**

The sero- and feco-prevalence of *H. pylori* infection was 62% and 51.1%, respectively. In those patients who had a positive result with either the antibody (Ab) or antigen (Ag) test, rural residence (AOR = 5.55; 95% CI: 2.34–13.14; *p* < 0.001), alcohol consumption (AOR = 12.34; 95% CI: 2.29–66.51; *p*=0.003), having no awareness about *H. pylori* transmission (AOR = 4.76; 95% CI: 1.86–12.15; *p*=0.001), using pond/river as a source of drinking water (AOR = 5.22; 95% CI: 1.91–14.27; *p*=0.001), and open field defecation (AOR = 4.19; 95% CI: 1.67–10.52; *p*=0.002) were the explanatory risk factors significantly associated with *H. pylori* infection.

**Conclusion:**

This study showed that the prevalence of *H. pylori* infection was very high. Most cases of gastric cancers are ascribed to *H. pylori* infection. Therefore, education should be given to communities so as to increase their awareness of the infection and the associated major explanatory risk factors identified in the present study.

## 1. Introduction

*Helicobacter pylori* (*H. pylori*) is a Gram-negative, microaerophilic rod-shaped bacterium with two to six lophotrichous flagella [1]. Numerous elements have been implicated as contributing factors for *H. pylori's* colonization and survival in the host's hostile stomach environment. Among these factors, but are not limited to, include (a) the presence of flagella, which enables the bacteria to move through and colonize the mucus layer of the gastric epithelium, (b) the ability of the bacteria to produce enzymes that dissolve the surfactant layer covering the gastric epithelium, and (c) the ability of the bacteria to produce large amounts of urease, which helps to neutralize the harsh acidic environment of the gastric mucosa by hydrolyzing urea to ammonia and carbon dioxide [2, 3].

*Helicobacter pylori* is an important pathogen, which is linked to a heavy burden of both malignant and nonmalignant disorders. The bacterium is classified as a human carcinogen and is highly linked to stomach cancer, which is the third most prevalent cancer-related death worldwide. It is also linked to disorders, including dyspepsia and peptic ulcer [4].

There are noticeable disparities in the epidemiology of *H. pylori* infection, particularly in children, between poor and developed countries [5]. *Helicobacter pylori* continues to be a significant global health issue, contributing significantly to morbidity and mortality from stomach cancer and peptic ulcer disease. Although it is estimated that half of the world's population has *H. pylori* infection, the prevalence of infection varies greatly between and within nations. Rapid economic development in developed nations, which boosted social standards, has been associated with a decline in infection rates. Despite the descending trend in prevalence worldwide, the overall prevalence in Africa remains very high (70%) [6]. However, these rates are more pronounced in children than in younger adults [7].

A previous systematic review and meta-analysis found out significant regional heterogeneity of *H. pylori* infections worldwide. It was shown that Oceania had the lowest prevalence (24.4%) compared to Africa which had the highest pooled prevalence (70.1%) of the infection [8]. A similar study on *H. pylori* infections further showed that Africa having the greatest global frequency of *H. pylori* infection [9].

In Ethiopia, the epidemiology of *H. pylori* exhibits striking heterogeneity across regions, study population, environmental factors, and lifestyles. In a meta-analysis study, the overall pooled prevalence of *H. pylori* infection was found to be 52.2%, with the Somali region having the highest prevalence (71%), followed by the Amhara region with a prevalence of 54.6% [10]. The prevalence of *H. pylori* varied across geographical locations in Ethiopia; it was 42.8% in Mizan Aman town, Southwest Ethiopia [11], 72% among gastritis patients attending Yekatit 12 Teaching Hospital of Addis Ababa [12], 47.7% in Bekoji [13], 30.3% in Shashamane, Ethiopia [14], and 65.7% in Ziway, central Ethiopia (65.7%) [15]. The frequency of *H. pylori* infection is also very high in the Amhara Regional State, but it is varied in different locations in the region. For example, the overall magnitude of *H. pylori* infection among dyspeptic patients attending Dessie Referral Hospital was 30.4% [16], 70.25% in Mekane Selam [17], and 34% in Debre Tabor [18].

Varied socioeconomic and hygienic conditions have been significantly associated with different prevalence rates of *H. pylori* infection, with individuals living in developed urban areas (less than 40%) being less infected than those living in rural developing areas (>80%) [19]. The population's sociodemographics, socioeconomic position, personal cleanliness, and way of life all have an impact on the prevalence of *H. pylori*. Some of the risk factors for *H. pylori* infection include crowding, unsanitary environments, alcohol use, the type of toilet facility, and unreliable water supplies [20, 21].

Serology tests are usually beneficial for sero-epidemiological studies, but not for individual patient decision-making. In addition, they need to be confirmed for particular sites and evaluated for erroneous results emanating from cross-reactivity of antigens from various pathogens. The accuracy of these tests might not go above 50% in areas with moderate *H. pylori* prevalence. However, the *H. pylori* stool antigen (HpSA) test is a more accurate way to identify recent and current *H. pylori* infection. In a single noninvasive test, the HpSA test has the ability to simultaneously test for antimicrobial resistance and give a speedy diagnosis [7].

According to Adet Primary Hospital's annual report, dyspepsia was ranked first from the top 10 diseases in the year 2021. Even though several studies have been conducted in different parts of Ethiopia, there has not been any study about sero- and feco-prevalence and associated risk factors of *H. pylori* infection among adult dyspeptic patients visiting Adet Primary Hospital, Yilmana Densa District. The research addressed the following research questions: what was the sero- and feco-prevalence of *H. pylori* infection among patients visiting Adet rimary Hospital? What were the major risk factors of *H. pylori* infection in the study area? What was the degree of association between the prevalence of *H. pylori* and potential risk factors? The specific objective of the present study was to determine the sero- and feco-prevalence, to identify the major explanatory risk factors, and also to determine the degree of association between the prevalence and the potential risk factors of *H. pylori* infection.

## 2. Materials and Methods

### 2.1. Study Area, Study Population, and Study Design

**Figure 1:**
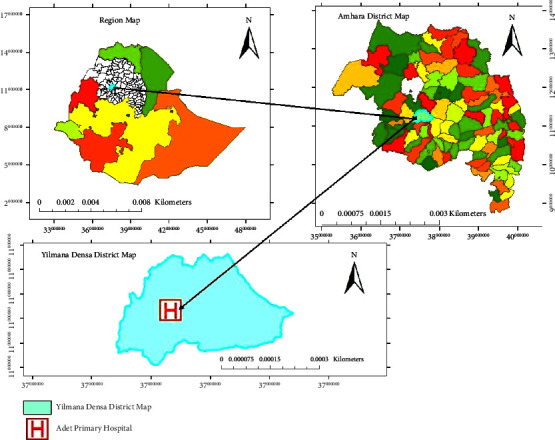
Location map of the study area.

Yilmana Densa District is located at a distance of 470 km from Addis Ababa (the capital city of Ethiopia) and 42 km away from Bahir Dar (the center city of the regional government). The district is located at the geographical location of 11° 29′ 60″ N latitude and 37° 19′ 60″ E longitude (Figure 1). The topography of the district is 56% plain, 20% mountain, and 23% lowland (depression), and 1% is occupied by water bodies. Its elevation ranges from 1500 to 2523 meter above sea level. The district is divided into the following three climatic zones: 64% Wayna Daga, 24% Dega, and 12% Kola. The mean annual rainfall ranges from 822 mm to 2000 mm and the average range of temperature is 14°C–27°C. The total population of the district in the year 2022, a projection based on the Ethiopian Census, was 228,725 of whom 111,120 were men and 117,605 were women. Of which, 26,198 or 11.5% were urban inhabitants and the rest 88.5% were rural inhabitants [22].

There are 11 health centers, five private clinics, 40 health posts, and one primary hospital in the Woreda. Adet Primary Hospital is in the study area and serves 60,697 (male 33,345 and female 27,352) populations in the catchment area. A hospital-based cross-sectional study among adult dyspeptic patients visiting the Outpatient Department (OPD) of Adet Primary Hospital was conducted from February 10, 2022, to April 10, 2022.

### 2.2. Sample Size Determination

The sample size (*n*) was calculated using the single population formula for cross-sectional surveys. Since there was no previous study conducted in the area, a 50% prevalence rate of *H. pylori* infection was taken, assuming that *H. pylori* is prevalent among patients in Adet Primary Hospital. The sample size was calculated with a 95% confidence interval and a 5% margin of error [23] as follows:

(1) $$\begin{array}{c} n=\frac{z^{2}p\left(1-p\right)}{d^{2}}\text{,} \end{array}$$

*z* is the critical value of the standard normal distribution, which is 1.96 at 95% CI, *p* is the estimated prevalence of *H. pylori* infection, and *d* (5%) is absolute precision.

Based on the abovementioned formula, a sample size of 384 was obtained. To compensate for the nonrespondents and minimize errors probably arising from the likelihood of noncompliance, 5% was added. Therefore, the final sample size for the study involved 403 study participants.

### 2.3. Sampling Techniques

The study participants were selected using a systematic random sampling technique. On average, at Adet Primary Hospital, 20 adult dyspeptic patients attended the outpatient department each day. Therefore, in the 22 working days of the month, there were 440 dyspeptic patients, and for the two months, there were 880 dyspeptic patients who requested participation in the study. The total expected number of dyspeptic patients during the study period was estimated to be 880. When the total population was divided by the sample size, the sample interval was two, and every two adult dyspeptic patients were selected until a total of 403 samples were obtained.

After obtaining written consent from each participant, a systematic random sampling technique was employed. Therefore, to get the required final sample size, the study participants were invited to take part in the study proportionally based on the number of dyspeptic patients who visited the outpatient department (OPD) during the study period. Thus, *k* = *N*/*n*, where “*k*” is the sample interval, “*N*” is the study population, and “*n*” is the sample size of the study =>880/403 = 2, and then the data were collected for every two adult dyspeptic patients who visited the OPD during data collection priorities.

### 2.4. Eligibility Criteria

#### 2.4.1. Inclusion Criteria

Those volunteers who signed the informed consent (18 years old and above) and also those who did not receive any anti-*H. pylori* treatment within the last four weeks prior to sampling were included.

#### 2.4.2. Exclusion Criteria

Those patients who did not sign the informed consent, who were less than 18 years old, and those patients treated with any antibiotics within the last four weeks of study enrollment were excluded from the study.

### 2.5. Variables of the Study

#### 2.5.1. Dependent Variable

The prevalence of *H. pylori* among adult dyspeptic patients.

#### 2.5.2. Independent Variables

All the associated risk factors of *H. pylori* that contributed to the prevalence of *H. pylori* among adult dyspeptic patients in the study area. These include, but are not limited to, sociodemographic and socioeconomic variables, lifestyle, behavioral, environmental, and clinical characteristics of the study participants.

### 2.6. Data Collection and Processing

Before data collection, written informed consent was obtained from study participants. The data were collected using a pretested, structured, and self-administered questionnaire by trained data collectors. Prior to data collection, the questionnaire was administered to 5% of participants attending another health center other than Adet Primary Hospital with upper gastrointestinal symptoms to assess the clarity and understandability of the question. For participants who could not read and write, interviews were employed based on the structured questionnaire. The questionnaire was developed in the Amharic language (the local language). Then, the responses were translated back into English. The completed questionnaires were collected and checked for completeness.

The blood and stool specimens were collected from each patient for *H. pylori* antigen and antibody tests, respectively, and immediately taken to the laboratory for processing. Some portion of the collected blood was used to determine the ABO and Rh blood groups by slide agglutination test using monoclonal anti-A, anti-B, anti-AB, and anti-D (Rh) antibodies according to the manufacturer's recommendation.

Some portion of the collected blood was allowed to clot in a test tube and centrifuged at 3000 rotations per minute for 10 minutes, and the serum was then separated. The separated serum was examined serologically for *H. pylori* immunoglobulin G (IgG) antibodies using immune chromatographic rapid test kits (dBest *H. pylori* test kit, Ameritech Diagnostic Reagent Co., Ltd., Tongxiang, Zhejiang, China), which are nationally approved and used for serological diagnosis of *H. pylori* infection. This test contains a membrane strip, which is precoated with *H. pylori* capture antigen on the test binding region. The *H. pylori* antigen-colloid gold conjugate and serum sample move along the membrane chromatographically to the test region (*T*) and form a visible line as the antigen-antibody-antigen gold particle complex forms. This test device has a letter of *T* and *C* as “Test Line” and “Control Line” on the surface of the case. Both the test line and control line in the result window are not visible before applying any samples. The control line is used for procedural control. The control line should always appear if the test procedure is performed properly and the test reagents in the control line are working. The test was performed following the manufacturer's instructions.

Study subjects with dyspepsia were provided with a clean, dry, leak-proof, disinfectant-free, and wide-mouthed plastic container to collect about 10 g of fresh stool specimens into the container for the *H. pylori* antigen test. Instructions were given on how to prevent contamination of the stool with water and urine. Immediately after collection of stool specimens, *H. pylori* antigens in stools were detected against the SD BIOLINE test strip that utilized a monoclonal anti-*H. pylori* antibody conjugate based on a lateral flow chromatographic immunoassay technique. Specimens were tested using the SD BIOLINE stool antigen test strip (Zhejiang Orient Gene Biotech Co, Ltd, China) with 94.9%–100% sensitivity and 95–100% specificity, according to the manufacturer's instructions. The stool sample was transferred to a vial with diluents, vigorously agitated, and after two minutes of resting the tube, dropped around two to three drops (80 mL) into the round window of the test cassette. Reading was performed after 10 minutes of incubation at room temperature, and based on the appearance of colored lines across the central window of the cassette, two lines, *C* (control) and *T* (test), indicate a positive test; only one line in C indicates a negative result. A pale-colored line in *T* is also considered positive.

### 2.7. Data Analysis

Data were analyzed using SPSS version 26.0 (SPSS Inc, Chicago, IL, USA). Descriptive statistics were used to characterize data and assess the distribution of study variables. Categorical variables were summarized in frequencies or percentages. A Chi-square test was performed to check for the presence of an association between dependent variables and independent variables. Univariate logistic regression was also carried out to identify and select variables with *p* < 0.25, which were candidates for the multivariate logistic regression model [24]. A multivariate logistic regression model was used after adjusting for confounding variables to identify the major explanatory risk factors for *H. pylori* infection among study participants. Variables with a *p* value <0.05 in the final model of multivariate logistic regression were considered statistically significant.

### 2.8. Ethical Considerations

Before conducting the investigation, the investigator obtained ethical clearance (Ref. No., PRCSVD/291/2014) from the Ethical Clearance Committee of Science College, Bahir Dar University. A letter describing the objective of the research was written to Adet Primary Hospital. Then, authorities at Adet Primary Hospital were informed and asked for permission. An informed, voluntary, written, and signed consent was obtained from the study subjects after explaining the importance of the study briefly. Participation in the study was on a voluntary basis, and participants were informed about their full right to withdraw or refuse at any stage of the study if they did not want to participate. Moreover, the confidentiality of the study participants was also maintained. Dyspeptic patients were tested for *H. pylori* following the national standard diagnosis guideline. Finally, the study participants whose test results were positive were treated with standard drugs as prescribed by a physician from Adet Primary Hospital.

## 3. Results

### 3.1. Sociodemographic and Socioeconomic Characteristics of Study Participants

A total of 403 study subjects participated in this study. The response rate was 100%. Among them, 210 (52.1%) were males and 193 (47.9%) were females. The age of the participants ranged from 18 to 64 years, with a mean and standard deviation of 38 ± 11.2 years. The majority (264; 65.5%) of the study participants were rural dwellers. More than half of the study participants (250; 62%) were illiterate. Two hundred twenty-nine (56.8%) of the study participants were married and 68 (16.9%) were single. More than half of the study participants (214; 53.1%) were farmers and 16 (4%) were students. Of the total, 116 (28.8%), 200 (49.6%), and 87 (21.6%) came from a family size of >5, 4–5, and ≤3, respectively. Two hundred thirty-two (57.6%) of the study participants had a monthly income of 1000–2500 Ethiopian Birr (ETB) and 88 (21.8%) had a monthly income below 1000 ETB (Table 1).

### 3.2. Clinical Characteristics of the Study Participants

With regard to blood group, 72 (17.9%) of the study participants had blood group A, 92 (22.8%) had blood group B, 102 (25.3%) had blood group AB, and 137 (34%) had blood group O. Majority of the study participants (248 (61.5%) were Rh positive. The highest proportion of the study participants had body mass index (BMI) category of normal (*n* = 196, 48.6%), followed by undernourished (*n* = 82, 20.3%), overweight (*n* = 71, 17.6%), and obese (*n* = 54, 13.4%).

Of the total study participants, 226 (56.1%), 212 (52.6%), 225 (55.8%), 216 (53.6%), 206 (51.1%), and 223 (55.3%) had histories of epigastric pain, heartburn, abdominal fullness, nausea, belching, and melena, respectively. However, only 199 (49.4%) and 112 (27.8%) had histories of vomiting and bloody vomiting, respectively (Table 2).

### 3.3. Sero- and Feco-Prevalence of *H. pylori* across Sociodemographic andSocioeconomic Variables

The highest prevalence of *H. pylori* infection, as determined by either Ab (antibody) or Ag (antigen) test, was observed in males (72.4%), in the age group of above 50 years (68.8%), rural residents (82.2%), illiterates (67.6%), married ones (66.8%), farmers (67.8%), family size of >5(67.2%), and in the study participants having a monthly income below 1000 ETB (71.6%) (Table 3). The 95% confidence interval for prevalence estimate is given as supplementary document (Table S1).

### 3.4. Sero- and Feco-Prevalence of *H. pylori* Infection

Out of the 403 examined adult dyspeptic patients, the number of positive patients who were detected by the serological test was 250, i.e., a sero-prevalence of 62%. The number of individuals who were found positive by serological test and became negative by stool antigen test was 55 (13.6%).

**Figure 2:**
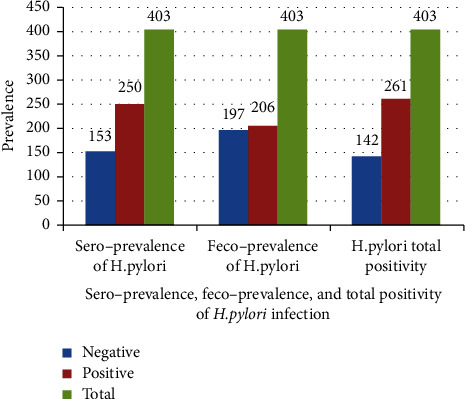
Sero- and feco-prevalence of *H. pylori* infection among dyspeptic patients in Yilmana Densa district, northwest Ethiopia, (*n* = 403).

Out of the total examined patients, the number of positive patients detected by the stool antigen test was 206 (51.1%), i.e., the feco-prevalence was 51.1%. The number of individuals who were found positive by stool antigen test and became negative by the serological test was 11 (2.7%). The number of positive patients who were detected by both serological and stool antigen test was 195 (48.4%). From the total, the number of individuals who were positive for either Ab or Ag test was 261 (64.8%) (Figure 2).

### 3.5. Risk Factors Associated with *H. pylori* Infection

In those patients who were positive for either Ab or Ag test, the univariate logistic regression analysis showed that male participants were 2.02 times (COR = 2.02; 95% CI: 1.33–3.06; *p* < 0.001) more likely to be infected by *H. pylori* than their female counterparts. Rural dwellers were 9.97 times (COR = 9.97; 95% CI: 6.19–16.06; *p* < 0.001) more likely to be infected with *H. pylori* than urban dwellers (Table 4).

Regarding alcohol consumption, those who consumed alcohol were 4.36 times (COR = 4.36; 95% CI: 2.78–6.83; *p* < 0.001) more likely to be infected with *H. pylori* infection than those who did not consume. Concerning smoking, those who smoke tobacco were 2.26 times more likely to be infected with *H. pylori* (COR = 2.26; 95% CI: 1.01–5.05; *p*=0.048). Study participants who did not have awareness about the transmission mode of *H. pylori* were 2.7 times more likely to be infected with *H. pylori* (COR = 2.7; 95% CI: 1.69–4.31; *p* < 0.001) than those who had awareness.

Regarding the source of water, those who used a pond/river as a source of drinking water were 7.73 times (COR = 7.73; 95% CI: 4.46–13.39; *p* < 0.001) at higher risk of *H. pylori* infection. Study participants who practiced open-field defecation were 5.05 times at higher risk of catching *H. pylori* infection (COR = 5.05; 95% CI: 3.04–8.40; *p* < 0.001) than those who had an improved latrine pit. The odds of *H. pylori* infection were about 4.65 times (COR = 4.65; 95% CI: 2.56–8.45; *p* < 0.001) higher among dyspeptic patients who did not wash their hands after toilet than those who washed their hands. Those dyspeptic patients who had low sanitary habits at home were 5.23 times at higher risk of acquiring *H. pylori* infection than those who had high sanitary habits at home (COR = 5.23; 95% CI:3.07–8.90; *p* < 0.001) (Table 4).

Variables with *p* values of <0.25 in univariate analysis were selected and entered into the multivariate logistic regression model. The sero-prevalence of *H. pylori* infection was significantly associated with alcohol consumption (AOR = 10.29; 95% CI: 1.59–66.59; *p*=0.014), lack of awareness about *H. pylori* transmission (AOR = 4.51; 95% CI: 0.99–20.57; *p*=0.05) and open field defecation (AOR = 5.46; 95% CI: 1.34–22.18; *p*=0.018). Rural residence (AOR = 4.2; 95% CI = 0.1–17.67; *p*=0.05) and source of drinking water (AOR = 11.03; 95% CI = 2.62–46.39; *p*=0.001) were significantly associated with the feco-prevalence of *H. pylori* infection.

In the final model of multivariate logistic regression, five independent variables, i.e., residence, alcohol consumption, awareness about the transmission mode of *H. pylori*, source of drinking water, and type of toilet facility, remained significantly associated with *H. pylori* infection, and they were found to be significant explanatory risk factors (*p* < 0.05) for *H. pylori* infection among dyspeptic patients during the study period (Table 4).

After adjusting the confounding variables in the multivariate analysis, rural dwellers were 5.55 times more likely to acquire *H. pylori* infection than urban dwellers (AOR = 5.55; 95% CI: 2.34–13.14; *p* < 0.001). Those who consumed alcohol had 12.34 times higher risk of acquiring *H. pylori* infection than their counterparts (AOR = 12.34; 95% CI: 2.29–66.51; *p*=0.003). The odds of occurrence of *H. pylori* infection among adult dyspeptic patients who did not have awareness on *H. pylori* transmission were about 4.76 times higher than those dyspeptic patients who had awareness (AOR = 4.76; 95% CI: 1.86–12.15; *p* < 0.001). The odds of *H. pylori* infection were about 5.22 times (AOR = 5.22; 95% CI: 1.91–14.27; *p* < 0.001) higher among dyspeptic patients who used pond/river as a source of drinking water than those who used tap water. Likewise, those who defecated in the open field were four times more likely to be infected with *H. pylori* (AOR = 4.19; 95% CI: 1.67–10.52; *p*=0.002) than their counterparts having improved latrines (Table 4).

In the final multivariate regression, it was found that males were 1.89 times at higher risk of *H. pylori* infection (AOR = 1.89; 95% CI: 0.86–4.15; *p*=0.111) than females, even though, it was not statistically significant. Even though it was not a significant explanatory risk factor, those participants who were above 50 years old were 1.61 times at higher risk of acquiring *H. pylori* infection (AOR = 1.61; 95% CI: 0.44–5.88, *p*=0.469). The odds of occurrence of *H. pylori* infection among adult dyspeptic patients who had a frequency of alcohol consumption >3 times per week were about 2.63 times higher than those dyspeptic patients who consumed alcohol only once per week. Nevertheless, alcohol consumption was not a statistically significant risk factor among the studied subjects (AOR = 2.63; 95% CI: 0.86–8.06; *p*=0.091). Although smoking was not found as one of the significant explanatory risk factors, smokers were 3.09 times at higher risk of acquiring *H. pylori* than their counterpart nonsmokers (AOR = 3.092; 95% CI: 0.45–19.17; *p*=0.225). Those who did not wash their hands after toilet were 2.78 times at higher risk of catching *H. pylori* infection (AOR = 2.78; 95% CI: 0.91–8.48; *p*=0.072). The odds of *H. pylori* infection were about 1.59 times higher among dyspeptic patients who had low sanitary habits at home (AOR = 1.59; 95% CI: 0.54–4.62; *p*=0.399). However, the level of sanitary habits was not a significant explanatory risk factor for *H. pylori* infection (Table 4).

## 4. Discussion

In the present study, the overall sero-prevalence of *H. pylori* infection among adult dyspeptic patients who visited the Adet Primary Hospital was 64.8% (261/403). It was in agreement with the findings from Cameron (64.39%) [25], Dessie Referral Hospital, northwest Ethiopia (60.5%) (16), Sudan (60%) [26], Pakistan (66.4%) [27], Iraq (62%) [28], and Kazakhstan (62.7%) [29]. It was higher than that of studies in Uganda (27.3%) [30], Yemen (29.99%) [31], Eritrea (31%) [32], South Korea (32.7%) [33], Lebanon (34.2%) [34], Canada (36%) [35], Libya (37%) [36], Romania (40.8%) [37], Japan (42.6%) [38], Mizan Aman (Ethiopia) (42.8%) [11], Vietnam (48.8%) [39], Korea (51%) [40], and Nigeria (52%) [41]. However, it was lower than the findings from Egypt (76.8%) [42], Nigeria (72.4%) [43], Egypt (72%) [44], Gonder University Hospital (Ethiopia) (71.1%) [45], Mekane Selam (Ethiopia) (70.25%) [17], Alaska, USA (68%) [46], and Zimbabwe (67.7%) [20]. These differences might be due to the differences in the titer of antibody and its persistence in study participants, diagnostic kits and techniques used, personal hygiene, degree of sanitation, sample size, water sources, sociodemographical and economical, and study setting (e.g., in our case hospital-based study targeting adult dyspeptic patients).

The overall feco-prevalence of *H. pylori* infection in this study was 51.1%. It is in line with findings from Hosaena town of southeast Ethiopia (51.4%) [47], Uganda (47.7%) [48], Bekoji (southeast Ethiopia) (47.7%) [13], Egypt (53.1%) [49], Iraq (54%) [50], and Nigeria (55%) [51]. It was lower than studies conducted in Sudan (70%) [26], Sekota (northeast Ethiopia) (64.8%) [52], and Pakistan (66.66%) [53]. In contrast, the findings of the current study were higher than reports from Debre Tabor (Ethiopia) (34%) [18], Addis Ababa Ethiopia (25.9%) [12], Nepal (17%) [54], Ghana (14.2%) [55], United Arab Emirates (41%) [56], and Iceland (3.4%) [57]. The differences in findings among various studies can be explained by variations in sociodemographic, socioeconomic, behavioral, types of diagnostic kits having different sensitivities, and environmental factors, as well as variations in the number of study population.

Rural residents had 5.55 times higher odds of *H. pylori* infection than their counterparts in urban areas. A similar study, which was conducted in Assosa (Ethiopia) [58], Alaska, U.S.A. [46], Addis Ababa (Ethiopia) [12], and Pakistan [59], identified this variable to be significantly associated with *H. pylori* infection. However, studies conducted in Dessie (Ethiopia) [16], Iraq [50], and Egypt [60] showed that residence was not significantly associated with *H. pylori* infection. The findings of the current study clearly showed that poor sanitary practice and a lack of access to clean water are associated with higher odds of *H. pylori* infection in rural residents than in urban residents.

The result of this study also showed that the odds of *H. pylori* infection were about 12.34 times higher among dyspeptic patients who consumed alcohol than those who did not consume alcohol. This finding is in agreement with studies conducted in Uganda (21), Bekoji (southeast of Ethiopia) [13], Cameron [61], Shashamane (Ethiopia) [14], Dessie (Ethiopia) [16], Indonesia [62], and Hosaena (Ethiopia) [47]. It is highly likely that alcohol can directly damage the gastric mucosal layer, favouring the attachment of the bacterium to the wall of the stomach. Moreover, frequent and heavy drinkers can possibly predispose them to social contacts that favor transmission of *H. pylori* infection.

Those adult dyspeptic patients who did not have awareness about the transmission of *H. pylori* infection were 4.76 times more likely to be at higher risk of acquiring *H. pylori* infection than those who had awareness. This might be due to the fact that those who do not have awareness of the transmission of *H. pylori* infection may not take appropriate preventive measures to protect themselves from the infection. Lack of awareness means there has been no action taken to avoid the infection. However, in contrast to the finding of the current study, a previous study conducted on the prevalence of *H. pylori* infection and its associated risk factors among patients undergoing upper gastrointestinal diagnosis in Shashamane Referral Hospital in Shashamane, Ethiopia, reported that awareness about *H. pylori* infection was not a significant risk factor associated with *H. pylori* infection [14].

Likewise, the multivariate analysis of this study also showed that the odds of *H. pylori* infection were about 5.22 times higher among dyspeptic patients who used a pond/river as a source of drinking water. Most people in the study area who did not have toilets might have defecated in the open field, which could contaminate water bodies used for drinking and food preparation, thereby increasing the chance of acquiring an *H. pylori* infection. This finding is in line with that of studies conducted in Uganda [63], Mizan Aman (Ethiopia) [11], Mekane Selam (Ethiopia) [17], Gondar University Hospital (Ethiopia) [45], Cameroon [61], Sekota (Ethiopia) [52], United Arab Emirates [56], Egypt [42], Pakistan [64], Alaska, U.S.A. [46], Nigeria [43], Debre Tabor (Ethiopia) [18], and Yemen [65].

Study participants who practiced open field defecation were 4.19 times at higher risk for *H. pylori* infection than those who used an improved latrine. These higher odds of infection might be associated with not having a toilet, leading them to defecate in the open field and at the same time exposing themselves and others for the acquisition of the bacterium from the open field. This finding is in agreement with studies conducted in Mizan Aman (Ethiopia) [11], Debre Tabor (Ethiopia) [18], and Ziway (Ethiopia) [15]. In contrast to the finding of the present study, the type of toilet used, however, was not significantly associated with *H. pylori* infection in studies conducted at Jasmine Internal Medicine and Pediatrics Specialized Private Clinic in Addis Ababa city (Ethiopia) [66].

Many previous studies reported that factors, such as family size [11, 16, 18, 63], lack of sanitary facilities at home [63], marital status [14], age group [14, 43, 56, 67], gender [11, 17, 47, 56, 58] monthly income, educational status [43, 66], sharing beds with siblings [11, 16], presence of domestic animals, storing and reusing water, and occupational status [11], were significantly associated with *H. pylori* infection. However, the current study did not show any significant association between *H. pylori* infection and these potential risk factors.

Generally, the findings of the present study could provide information that can be utilized in the planning of meaningful public health control programs in the prevention and control of *H. pylori* infection. Furthermore, it also provides information on the major explanatory risk factors of *H. pylori* infection in the study area.

## 5. Limitation of the Study

This study was limited only to adult dyspeptic patients attending the outpatient department of the Adet Primary Hospital from February 10 to April 10, 2022. The study did not include adult dyspeptic patients visiting other health centers in the district. The employment of a rapid serology (IgG) test kit for the diagnosis could not detect active *H. pylori* infection because high-titer IgG can last for months to years. This may overestimate the prevalence of *H. pylori* infection among the study participants since the total positivity was determined by the combination of the two tests. Moreover, this study was limited only to symptomatic patients visiting the hospital. Therefore, it might not show the actual prevalence of the infection in the entire population of the district.

## 6. Conclusion

This study revealed a high (64.4%) overall prevalence of *H. pylori* infection among adult dyspeptic patients attending the Adet Primary Hospital. The sero- and feco-prevalence of *H. pylori* infection in this study were 62% and 51.1%, respectively. Rural residence, lack of awareness on *H. pylori* transmission, alcohol consumption, using a pond/river as a source of drinking water, and open field defecation were found to be significant explanatory risk factors associated with *H. pylori* infection among study participants. However, none of the clinical characteristics considered in the present study were significantly associated (*p*>0.05) with *H. pylori* infection among the studied subjects.

The present findings have potential implications for clinical practice and public health in the study area. The very high prevalence of *H. pylori* infection among the studied population calls for certain intervention mechanisms (e.g., education) by concerned bodies, as most gastric cancer cases are associated with this particular infection. Although not statistically significant, close to 65% of study participants infected with *H. pylori* (Table 2) had a history of epigastric pain, heartburn, abdominal fullness, nausea, belching, melena, vomiting, and bloody vomiting. These clinical signs and symptoms can be used by clinicians to suspect *H. pylori* infection and accordingly order patients for the diagnosis of *H. pylori* active infection from stool specimens. At the community level, *H. pylori* infection and its effects (e.g., gastric cancer) and associated factors such as consumption of alcohol, source of drinking water, and type of toilet facility should be further studied to reduce the prevalence and transmission of *H. pylori*.

## Figures and Tables

**Table 1:** Sociodemographic and socioeconomic characteristics of the study participants in Yilmana Densa district, northwest Ethiopia, (*n* = 403).

Variables	Categories	Frequency	Percent
Gender	Male	210	52.1
	Female	193	47.9

Age (year)	18–29	90	22.3
	30–39	138	34.2
	40–49	95	23.6
	≥50	80	19.9

Residence	Rural	264	65.5
	Urban	139	34.5

Educational status	Illiterate	250	62
	Primary (1–8)	96	23.8
	Secondary (9–12)	42	10.4
	Diploma and above	15	3.7

Marital status	Married	229	56.8
	Widowed	48	11.9
	Divorced	58	14.4
	Single	68	16.9

Occupational status	Farmer	214	53.1
	Daily laborer	67	16.6
	Merchant	33	8.2
	Student	16	4
	Government employee	21	5.2

Family size	≤3	87	21.6
	4–5	200	49.6
	>5	116	28.8

Monthly income (ETB) of the family	Below 1000	88	21.8
	1000–2500	232	57.6
	Above 2500	83	20.6

**Table 2:** Clinical characteristics of the study participants in Yilmana Densa district, northwest Ethiopia, (*n* = 403).

Variables	Categories	*N* (percent)	*H.pylori* positive (%)	*H.pylori* negative (%)	*χ* ^2^(*p* value)
ABO group blood	A	92 (22.8)	54 (58.7%)	38 (41.3%)	3.249 (0.355)
	B	72 (17.9)	46 (63.9%)	26 (36.1%)	
	AB	102 (25.3)	65 (63.7%)	37 (36.3%)	
	O	137 (34)	96 (70.1%)	41 (29.9%)	

Rhesus (Rh) factor	Rh + ve	248 (61.5)	165 (66.5%)	83 (33.5%)	0.883 (0.347)
	Rh-ve	155 (38.5)	96 (61.9%)	59 (38.1%)	

BMI category (Kg/m2)	Undernourished	82 (20.3)	56 (68.3%)	26 (31.7%)	0.860 (0.835)
	Normal	196 (48.6)	125 (63.8%)	71 (36.2%)	
	Overweight	71 (17.6)	44 (62%)	27 (38%)	
	Obese	54 (13.4)	36 (66.7%)	18 (33.3%)	

Epi-gastric pain	Yes	226 (56.1)	148 (65.5%)	78 (34.5%)	0.118 (0.732)
	No	177 (43.9)	113 (63.8%)	64 (36.2%)	

Heart burn	Yes	212 (52.6)	138 (65.1%)	74 (34.9%)	0.021 (0.884)
	No	191 (47.4)	123 (64.4%)	68 (35.6%)	

Abdominal fullness	Yes	225 (55.8)	148 (65.8%)	77 (34.2%)	0.229 (0.632)
	No	178 (44.2)	113 (63.5%)	65 (36.5%)	

Vomiting	Yes	199 (49.4)	130 (65.3%)	69 (34.7%)	0.054 (0.815)
	No	204 (50.6)	131 (64.2%)	73 (35.8%)	

Nausea	Yes	216 (53.6)	141 (65.3%)	75 (34.7%)	0.054 (0.817)
	No	187 (46.4)	120 (64.2%)	67 (35.8%)	

Belching	Yes	206 (51.1)	135 (65.5%)	71 (34.5%)	0.109 (0.741)
	No	197 (48.9)	126 (64%)	71 (36%)	

Melena	Yes	223 (55.3)	146 (65.5%)	77 (34.5%)	0.109 (0.741)
	No	180 (44.7)	115 (63.9%)	65 (36.1%)	

Bloody vomiting	Yes	112 (27.8)	74 (66.1%)	38 (33.9%)	0.116 (0.733)
	No	291 (72.2)	187 (64.3%)	104 (35.7%)	

**Table 3:** Sero- and feco-prevalence of *H. pylori* infection across socio-demographic and-economic characteristics of study participants in Yilmana Densa district, northwest Ethiopia, (*n* = 403).

Socio-demographic and- economic variables	Total subjects examined	Positive for serology test *n* (%)	Positive for stool antigen test *n*(%)	Positive for either Ab or Ag test
*Gender*				
Male	210	145 (69)	123 (58.6)	152 (72.4)
Female	193	105 (54.4)	83 (43)	109 (56.5)

*Age (years)*				
18–29	90	51 (56.7)	42 (46.7)	52 (57.8)
30–39	138	90 (65.2)	74 (53.6)	94 (68.1)
40–49	95	57 (60)	47 (49.5)	60 (63.2)
≥50	80	52 (65)	43 (53.8)	55 (68.8)

*Residences*				
Rural	264	206 (78)	175 (66.3)	217 (82.2)
Urban	139	44 (31.7)	31 (22.3)	44 (31.7)

*Educational status*				
Illiterate	250	162 (64.8)	133 (53.2)	169 (67.6)
Primary (1–8)	96	57 (59.4)	51 (53.1)	59 (61.5)
Secondary (9–12)	42	25 (59.5)	16 (38.1)	25 (59.5)
Diploma and above	15	6 (40)	6 (40)	8 (53.3)

*Marital status*				
Married	229	145 (63.3)	122 (53.3)	153 (66.8)
Widowed	48	30 (62.5)	23 (47.9)	30 (62.5)
Divorced	58	36 (62.1)	32 (55.2)	38 (65.5)
Single	68	39 (57.4)	29 (42.6)	40 (58.8)

*Occupational status*				
Farmer	214	140 (65.4)	120 (56.1)	145 (67.8)
Daily laborer	67	39 (58.2)	28 (41.8)	41 (61.2)
Merchant	33	19 (57.6)	17 (51.5)	20 (60.6)
Student	16	9 (56.3)	8 (50)	10 (62.5)
House wife	21	14 (66.7)	8 (38.1)	14 (66.7)
Government employee	52	29 (55.8)	25 (48.1)	31 (59.6)

*Family size*				
≤3	87	54 (62.1)	45 (51.7)	55 (63.2)
4-5	200	120 (60)	102 (51)	128 (64)
>5	116	76 (65.5)	59 (50.9)	78 (67.2)

*Monthly income (ETB)*				
Below 1000	88	60 (68.2)	53 (60.2)	63 (71.6)
1000–2500	232	142 (61.2)	117 (50.4)	147 (63.4)
Above 2500	83	48 (57.8)	36 (43.4)	51 (61.4)

**Table 4:** Univariate and multivariate logistic regression analyses of potential risk factors associated with *H. pylori* in Yilmana Densa district, northwest Ethiopia, 2022.

Variables	Positive for serology test (%)	Positive for stool antigen test (%)	Univariate logistic regression		Multivariate logistic regression	
			COR (95% CI)	*p* value	AOR (95% CI)	*p* value
*Gender*						
Male	145 (69)	123 (58.6)	2.02 (1.33–3.06)	0.001^*∗*^	1.89 (0.86–4.15)	0.111
Female	105 (54.4)	83 (43)	1		1	

*Age (year)*						
18–29	51 (56.7)	42 (46.7)	1		1	
30–39	90 (65.2)	74 (53.6)	0.62 (0.33–1.17)	0.140	0.68 (0.23–2.02)	0.488
40–49	57 (60)	47 (49.5)	0.97 (0.54–1.76)	0.923	0.69 (0.20–2.35)	0.550
Above 50	52 (65)	43 (53.8)	0.78 (0.42–1.46)	0.438	1.61 (0.44–5.88)	0.469

*Residence*						
Rural	206 (78)	175 (66.3)	9.97 (6.19–16.06)	<0.001^*∗*^	5.55 (2.34–13.14)	<0.001^*∗*^
Urban	44 (31.7)	31 (22.3)	1			

*Marital status*						
Married	145 (63.3)	122 (53.3)	1.41 (0.81–2.46)	0.226	1.28 (0.45–3.62)	0.641
Widowed	30 (62.5)	23 (47.9)	1.17 (0.55–2.49)	0.69	1.94 (0.50–7.45)	0.335
Divorced	36 (62.1)	32 (55.2)	1.33 (0.64–2.75)	0.441	1.04 (0.28–3.84)	0.955
Single	39 (57.4)	29 (42.6)	1		1	

*Monthly income (ETB)*						
Below 1000	60 (68.2)	53 (60.2)	1.58 (0.83–3.00)	0.161	0.95 (0.29–3.10)	0.932
1000–2500	142 (61.2)	117 (50.4)	1.09 (0.65–1.82)	0.757	0.99 (0.38–2.57)	0.977
Above 2500	48 (57.8)	36 (43.4)	1		1	

*Alcohol consumption*						
Yes	200 (71.7)	168 (60.2)	4.36 (2.78–6.83)	<0.001^*∗*^	12.34 (2.29–66.51)	0.003∗
No	50 (40.3)	38 (30.6)	1		1	

*Frequency of alcohol consumption*						
Once/week	27 (58.7)	23 (50)	1		1	
1–3 times/week	77 (62.6)	60 (48.8)	0.71 (0.34–1.46)	0.35	1.27 (0.45–3.57)	0.648
>3 times/week	82 (74.5)	72 (65.5)	1.66 (0.76–3.60)	0.205	2.63 (0.86–8.06)	0.091

*Tobacco smoking*						
Yes	30 (76.9)	23 (59)	2.26 (1.01–5.05)	0.048^*∗*^	3.09 (0.45–19.17)	0.225
No	220 (60.4)	183 (50.3)	1		1	

*Khat chewing*						
Yes	40 (70.2)	38 (66.7)	1.62 (0.87–3.05)	0.131	1.20 (0.40–3.62)	0.748
No	210 (60.7)	168 (48.6)	1		1	

*Coffee drinking*						
Yes	162 (63.8)	135 (53.1)	1.29 (0.85–1.96)	0.235	1.39 (0.64–3.04)	0.411
No	88 (59.1)	71 (47.7)	1		1	

*Raw milk consumption*						
Yes	144 (64.9)	120 (54.1)	1.43 (0.95–2.16)	0.085	1.29 (0.59–2.83)	0.529
No	106 (58.6)	86 (47.5)	1		1	

*Awareness on transmission of H. pylori*						
Yes	46 (46.9)	32 (32.7)	1		1	
No	204 (66.9)	174 (57)	2.7 (1.69–4.31)	<0.001^*∗*^	4.76 (1.86–12.15)	0.001^*∗*^

*Source of drinking water*						
Piped water	76 (42.9)	58 (32.8)	1		1	
Spring water	55 (68.8)	47 (58.8)	3.69 (2.04–6.52)	<0.001^*∗*^	1.73 (0.63–4.73)	0.285
Pond/river	119 (81.5)	101 (69.2)	7.73 (4.46–13.39)	<0.001^*∗*^	5.22 (1.91–14.27)	0.001^*∗*^

*Type of toilet facility*						
Improved latrine pit	53 (42.1)	43 (34.1)	1		1	
Unimproved pit	56 (58.3)	44 (45.8)	1.85 (1.08–3.17)	0.026^*∗*^	1.14 (0.42–3.14)	0.794
Open field	141 (77.9)	119 (65.7)	5.05 (3.04–8.40)	<0.001^*∗*^	4.19 (1.67–10.52)	0.002^*∗*^

*Habit of hand washing after toilet*						
Yes	19 (33.3)	17 (29.8)	1		1	
No	231 (66.8)	189 (54.6)	4.65 (2.56–8.45)	<0.000^*∗*^	2.78 (0.91–8.48)	0.07

*Share beds with siblings*						
Yes	228 (63.2)	188 (52.1)	0.66 (018 3−1.19)	0.154	2.78 (0.91–8.48)	0.072
No	22 (52.4)	18 (42.9)	1		1	

*Sanitary practice at home*						
Low	109 (73.6)	94 (63.5)	5.226 (3.067–8.904)	<0.001^*∗*^	1.59 (0.54–4.62)	0. 399
Medium	90 (68.7)	71 (54.2)	3.266 (1.947–5.479)	<0.001^*∗*^	1.34 (0.50–3.54)	0.561
High	51 (41.11)	41 (33.1)	1		1	

## Data Availability

The data that support the findings of the study are available within the article and raw data can be obtained from the corresponding author upon request.

## Supplementary Materials

Supplementary Materials Table S1: Confidence intervals (95% CI) for sero- and feco-prevalence estimates of *H. pylori* infection.
