# Supplementary material for: Sero- and Feco-Prevalence of Helicobacter pylori Infection and Its Associated Risk Factors among Adult Dyspeptic Patients Visiting the Outpatient Department of Adet Primary Hospital, Yilmana Densa District, Northwest Ethiopia
Source: Can J Infect Dis Med Microbiol. 2023 Jul 17;2023:2305681. doi: 10.1155/2023/2305681 (PMC10365919; doi:10.1155/2023/2305681)
Supplement: Supplementary Materials — Table S1: Confidence intervals (95% CI) for sero- and feco-prevalence estimates of H. pylori infection. [file 2305681.f1.pdf]

Socio-demographic and- economic variables	Total subjects examined	Positive for serology test	Positive for stool antigen test	Positive for either Ab or Ag test
		n (%: 95% CI)	n (%:95% CI)	n(%:95% CI)
**Gender**				
Male	210	145 (69: 62.3-75.2)	123 (58.6: 51.6-65.3)	152 (72.4:65.8-78.3)
Female	193	105 (54.4:47.1-61.6)	83 (43:35.9-50.3)	109 (56.5:49.2-63.6)
Total	403	250 (62: 57.1-66.8)	206 (51.1: 46.1-56.1)	261(64.8:59.9-69.4)
**Age (year)**				
18-29	90	51 (56.7:45.8-67.1)	42 (46.7:36.1-57.5)	52 (57.8:46.9-68.1)
30-39	138	90 (65.2:56.6-73.1)	74 (53.6:44.9-62.1)	94 (68.1:59.6-75.8)
40-49	95	57 (60:49.4-69.9)	47 (49.5:39-59.9)	60 (63.2:52.6-72.8)
Above 50	80	52 (65:53.5-75.3)	43 (53.8:42.2-65.0)	55 (68.8:57.4-78.7)
Total	403	250(62: 57.1-66.8)	206 (51.1: 46.1-56.1)	261(64.8:59.9-69.4)
**Residences**				

Rural	264	206 (78:72.5-82.9)	175 (66.3:60.2-72.0)	217 (82.2:77.0-86.6)
Urban	139	44 (31.7:24.0-40.1)	31 (22.3:15.7-30.1)	44 (31.7:24.0-40.1)
Total	403	250(62: 57.1-66.8)	206(51.1: 46.1-56.1)	261(64.8:59.9-69.4)
**Educational status**				
Illiterate	250	162(64.8:58.5-70.7)	133(53.2:46.8-59.5)	169(67.6:61.4-73.4)
Primary(1-8)	96	57(59.4:48.9-69.3)	51(53.1:42.7-63.4)	59(61.5:51.0-71.2)
Secondary(9-12)	42	25(59.5:43.3-74.4)	16(38.1:23.6-54.4)	25(59.5:43.3-74.4)
Diploma and above	15	6(40:16.3-67.7)	6(40:16.3-67.7)	8(53.3:26.6-78.7)
Total	403	250(62: 57.1-66.8)	206(51.1: 46.1-56.1)	261(64.8:59.9-69.4)
**Marital status**				
Married	229	145(63.3:56.7-69.6)	122(53.3:46.6-59.9)	153(66.8:60.3-72.9)
Widowed	48	30(62.5:47.4-76.0)	23(47.9:33.3-62.8)	30(62.5:47.4-76.0)
Divorced	58	36(62.1:48.4-74.5)	32(55.2:41.5-68.3)	38(65.5:51.9-77.5)
Single	68	39(57.4:44.8-69.3)	29(42.6:30.7-55.2)	40(58.8:46.2-70.6)

Total	403	250(62: 57.1-66.8)	206(51.1: 46.1-56.1)	261(64.8:59.9-69.4)
**Occupational status**				
Farmer	214	140(65.4:58.6-71.8)	120(56.1:49.1-62.8)	145(67.8:61.0-74.0)
Daily laborer	67	39(58.2:45.5-70.2)	28(41.8:29.8-54.5)	41(61.2:48.5-72.9)
Merchant	33	19(57.6:39.2-74.5)	17(51.5:33.5-69.2)	20(60.6:42.1-77.1)
Student	16	9(56.3:29.9-80.2)	8(50:24.7-75.3)	10(62.5:35.4-84.8)
House wife	21	14(66.7:43.0-85.4)	8(38.1:18.1-61.6)	14(66.7:43.0-85.4)
Government employee	52	29(55.8:41.3-69.5)	25(48.1:34.0-62.4)	31(59.6:45.1-73.0)
Total	403	250(62: 57.1-66.8)	206(51.1: 46.1-56.1)	261(64.8:59.9-69.4)
**Family size**				
≤3	87	54(62.1:51.0-72.2)	45(51.7:40.7-62.6)	55(63.2:52.2-73.3)
4-5	200	120(60:52.9-66.8)	102(51:43.9-58.1)	128(64:56.9-70.6)
>5	116	76(65.5:56.1-74.1)	59(50.9:41.4-60.3)	78(67.2:57.9-75.7)
Total	403	250(62: 57.1-66.8)	206(51.1: 46.1-56.1)	261(64.8:59.9-69.4)

**Monthly income (ETB)**				
Below 1000	88	60(68.2:57.4-77.7)	53(60.2:49.2-70.5)	63(71.6:61.0-80.7)
1000-2500	232	142(61.2:54.6-67.5)	117(50.4:43.8-57.0)	147(63.4:56.8-69.6)
Above 2500	83	48(57.8:46.5-68.6)	36(43.4:32.5-54.7)	51(61.4:50.1-71.9)
Total	403	250(62: 57.1-66.8)	206(51.1: 46.1-56.1)	261(64.8:59.9-69.4)

Supplementary information for Table 3 in the manuscript

Table S1. Confidence intervals (95% CI) for sero- and feco-prevalence estimates of *H. pylori* infection across socio-demographic and -economic characteristics of study participants in Yilmana Densa District, northwest Ethiopia, (n=403).
